# Assessment of Facial Pain After Internal Carotid Artery Stenting: The Role of External Carotid Artery Overstenting

**DOI:** 10.3390/jcm13247666

**Published:** 2024-12-16

**Authors:** Dorota Łyko-Morawska, Łukasz Szkółka, Michał Serafin, Emila Senderek, Paweł Kiczmer, Magdalena Górska, Wacław Kuczmik

**Affiliations:** 1 Department of General Surgery, Vascular Surgery, Angiology and Phlebology, Faculty of Medical Sciences in Katowice, Medical University of Silesia, 45-47 Ziołowa Street, 40-635 Katowice, Poland; 2 Department of Pathomorphology, The Faculty of Medical Sciences in Zabrze, Medical University of Silesia, 3 Maja 13 Street, 41-800 Zabrze, Poland

**Keywords:** carotid artery stenting, internal carotid artery, external carotid artery, facial pain

## Abstract

**Background**: The external carotid artery (ECA) supplies blood to various facial and neck regions and may contribute to collateral cerebral perfusion. With the rise in carotid artery stenting (CAS) as a treatment for carotid stenosis, ECA overstenting has become a common procedure feature. This study aimed to assess the incidence, characteristics, and duration of facial pain following CAS, hypothesizing that ECA overstenting may contribute to facial pain. **Materials and Methods**: This prospective study included 55 patients treated with CAS for internal carotid artery (ICA) stenosis at a single center. Patients’ facial pain was evaluated using a numeric rating scale (NRS) before, immediately after, and 24 h post-CAS. Patient data, including demographics, comorbidities, and procedural details, were analyzed to assess the relationship between ICA stenosis degree, ECA diameter changes, and facial pain incidence. **Results**: CAS was associated with intraoperative facial pain in 27.27% of patients, with 7.28% reporting residual pain 24 h post-procedure. Pain occurrence was significantly higher in patients with lower ICA stenosis (*p* = 0.04). The median ECA diameter decreased from 4.11 mm to 3.16 mm (*p* < 0.001) after CAS, with ECA overstenting observed in 96.4% of cases. No significant relationship was found between pain severity and stent width or length. **Conclusions**: This study highlights that CAS significantly decreases the diameter of ECA. Additionally, ECA overstenting might be associated with perioperative and postoperative facial pain, emphasizing the need for careful monitoring of ECA patency following CAS.

## 1. Introduction

The external carotid artery (ECA) has eight main branches: six collateral branches (superior thyroid artery, lingual artery, facial artery, ascending pharyngeal artery, occipital artery, and posterior auricular artery) and two terminal branches (maxillary artery and superficial temporal artery). These branches deliver blood to the muscles of the neck, the upper larynx, the thyroid gland, the tongue and sublingual areas, and various parts of the face including the lips, nose, and ocular regions, as well as the occipital region, scalp, and ear. Additionally, the ECA supplies deep facial structures, such as the mandible, pterygoid region, and the infratemporal fossa [1,2]. Therefore, diseases of ECA such as stenosis or occlusion might be linked to the occurrence of facial pain [3].

Facial pain is a diagnostic and therapeutic challenge for clinicians as well as patients and it is associated with high morbidity and high levels of health care utilization. Unfortunately, these conditions are mostly regarded as a diagnosis of exclusion. The International Association for the Study of Pain classification (IASP) and the International Headache Classification (ICHD-III) include the diagnostic criteria for facial pain. Pain is defined as an unpleasant sensory and/or emotional experience according to the IASP [4,5]. Two types of pain can be distinguished: The first is receptor pain which is associated with an inflammation or injury of facial structures. The second type is neuropathic pain which occurs due to neural dysfunction [6,7]. Amongst the first type, the most common is toothache caused by enamel or dentin loss; other causes include temporo-mandibular joint dysfunction, sinusitis, or inflammation of the ears or salivary glands. Nonetheless, in this type of facial pain, the other symptoms of the underlying disease may be observed. The second type can be divided into two groups: the neuropathic pain and the neuralgic pain (neuralgia) [6]. Neuropathic pain results from the damage to a nerve or its branches; the damage may be caused by trauma, long-term metabolic disease (e.g., diabetes), or inflammation [8]. On the other hand, neuralgic pain often results from an unknown cause (e.g., trigeminal neuralgia) [1]. In addition, facial pain may have psychogenic and idiopathic causes [9]. A new, not fully investigated phenomenon has emerged due to the development of endovascular surgery. This phenomenon is associated with the occurrence of facial pain in patients treated with carotid artery stenting (CAS) for the stenosis of the carotid artery [10]. Facial pain can arise perioperatively (during or immediately after the procedure) or postoperatively, each with distinct clinical implications. Perioperative pain is often related to procedural factors, including mechanical irritation or ischemia during stent deployment (including acute ischemia due to the reduced blood flow in ECA), whereas postoperative pain may result from longer-term complications such as altered blood flow dynamics, stenosis progression, or neural involvement. Understanding both types is essential for timely diagnosis and management, as they may differ in etiology, duration, and therapeutic approach [10].

Carotid artery stenting (CAS) has become a less invasive alternative to carotid endarterectomy (CEA) in the treatment of carotid artery stenosis [3]. The traditional CEA is performed on both the internal carotid artery (ICA) and external carotid artery (ECA). On the other hand, CAS involves a deployment of the stent in the ICA that extends to the bulb of the common carotid artery (CCA); this prevents the recurrence of stenosis in the CCA bulb. However, in this situation, overstenting occurs, which is defined as the transverse crossing of the stent above the orifice of the ECA [11]. In vitro [12] as well as in vivo [13] studies have shown that the extension of the stent across the orifice of the ECA may result in abnormal blood flow in the artery. In addition, De Borst et al. in their study have reported the significant progression of stenosis in the ipsilateral ECA compared with the untreated contralateral ECA [11]. Additionally, some studies have reported that the occlusion of the ECA may result in facial pain, e.g., in the jaw claudication [14,15,16,17,18].

In addition, in recent years, carotid stent design (single-layer closed cell) has been linked to minor cerebral strokes due to the atherosclerotic plaque prolapsing through the stent struts resulting in cerebral embolism [19,20]. Therefore, the second-generation carotid stents (SGSs) have been developed.

First-generation stents (FGSs) and second-generation stents (SGSs) exhibit fundamental differences in their construction. FGSs are characterized by a simple single-layer design made entirely of nitinol, a superelastic alloy that provides flexibility, self-expansion, and durability. In contrast, SGSs utilize a more complex multi-layered design. While FGSs rely solely on nitinol for their structure, SGSs incorporate a combination of materials, such as nitinol, for the structural framework, and other materials, like polyethylene terephthalate (PET), for the mesh. Additionally, SGSs differ in the placement and design of their mesh. For instance, the mesh in SGSs may be braided, knitted, or fenestrated and can either wrap around the stent frame or be positioned inside it, offering enhanced plaque coverage, embolic protection, and improved adaptability to specific clinical needs [21,22]. Due to the differences in stent design between FGSs and SGSs, an additional factor contributing to abnormal blood flow in the external carotid artery (ECA) has emerged. The multi-layered structure and varied mesh configurations in SGSs, while improving plaque coverage and embolic protection, can alter the hemodynamic profile within the stented region. Factors such as mesh position, density, and the interaction between the stent frame and vessel wall can influence flow dynamics, potentially disrupting laminar blood flow and increasing the risk of turbulence or altered flow patterns in adjacent branches like the ECA.

Nonetheless, the facial pain occurring from the ECA stenosis or occlusion after CAS may be transient and resolved spontaneously [10]. The rising popularity of CAS and SGSs in the treatment of carotid artery stenosis can result in a growing population of patients with facial pain.

Given the limited and ambiguous information regarding this phenomenon, this study aims to investigate the incidence, characteristics, and duration of facial pain in patients undergoing CAS at the Department of General Surgery, Vascular Surgery, Angiology, and Phlebology at the Medical University of Silesia, Poland. The goal is to systematize knowledge and enhance awareness of this issue among healthcare professionals, including both doctors and dentists.

## 2. Materials and Methods

### 2.1. Study Design and Studied Population

The prospective study included all patients from the Department of General Surgery, Vascular Surgery, Angiology, and Phlebology at the Medical University of Silesia in Katowice, Poland, who were admitted to the department with the diagnosis of carotid artery stenosis between February 2023 and May 2023. The inclusion criteria were as follows: diagnosis of carotid artery stenosis via Doppler Ultrasonography, treatment with carotid artery stenting, and age above 18 years old. The exclusion criteria were as follows: treatment of carotid artery stenosis with carotid endarterectomy and lack of patient consent.

### 2.2. Pain Measurement

Before, immediately after, and 24 h following carotid artery stenting (CAS), patients assessed their facial pain levels using a numeric rating scale (NRS) based on the authors’ original survey. This survey was designed to capture the intensity of facial pain experienced by patients at these specific time points, allowing for a detailed evaluation of pain progression related to the procedure. The NRS provided a standardized method for patients to quantify their pain, facilitating a clearer understanding of the relationship between CAS and facial pain.

### 2.3. Equipment

The carotid artery angiography was performed with the Artis zee ceiling (Siemens Healthineers AG, Forchheim, Germany) system. During CAS, the 1st and 2nd generation carotid stents were used. The type as well as the length and width of the stent were chosen by the main vascular surgeon performing the procedure.

### 2.4. Analyzed Data

Patient data, including general characteristics (e.g., age, gender, comorbidities, and clinical symptoms), were gathered from electronic medical records within our department.

Details regarding carotid artery stenting, such as stent type, width, and length, were extracted from procedural reports.

The degree of ICA stenosis was assessed in preoperative angiogram using the NASCET method, following the guidelines of the European Society of Vascular Surgery [23] (Supplementary Materials).

**Figure 1:**
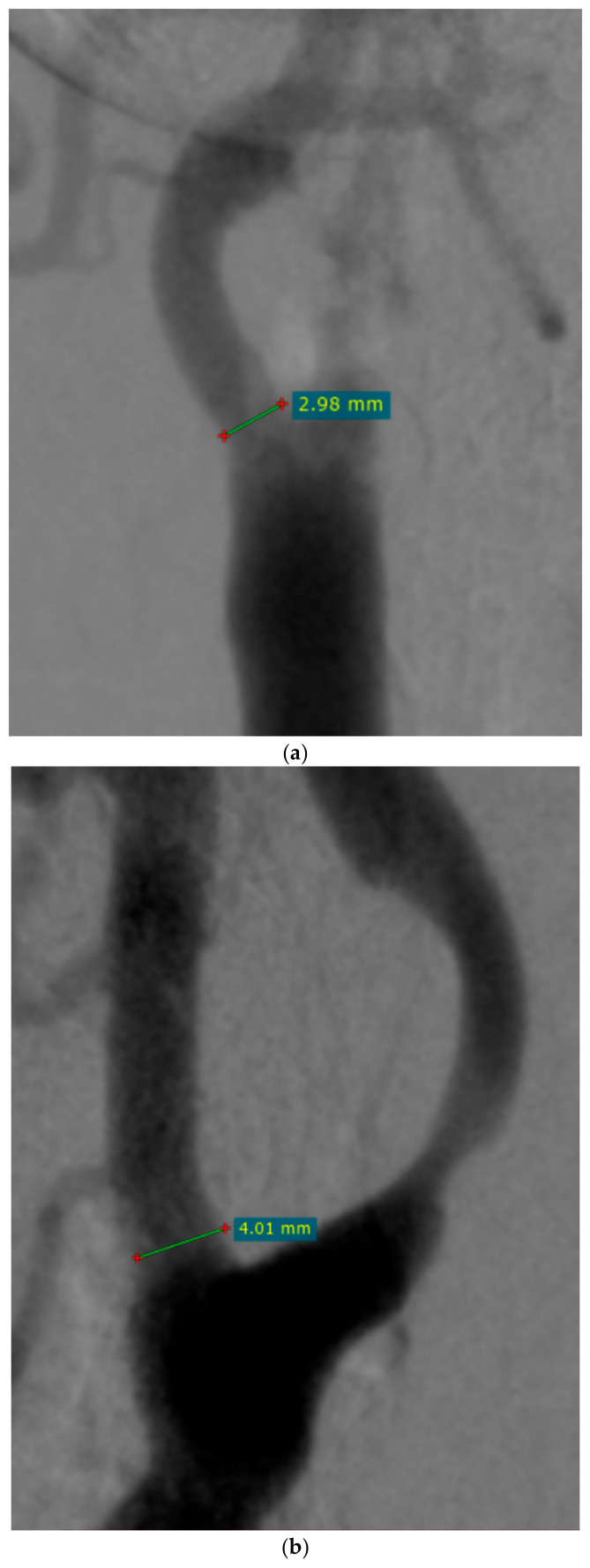
Pre-procedural angiograms with the measurements of external carotid artery (ECA) diameter (**a**) in a 69-year-old woman and (**b**) in a 76-year-old man (RadiAnt DICOM VIEWER, Medixant, Poznań, Poland).

**Figure 2:**
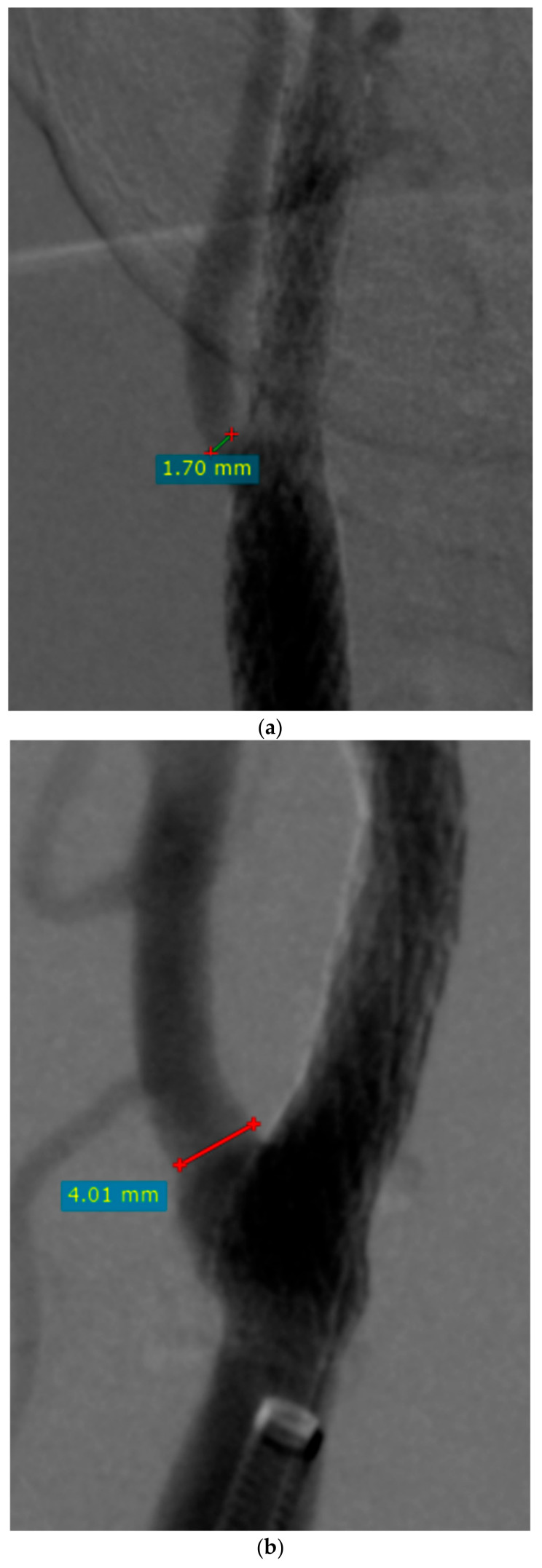
Post-procedural angiograms with the measurements of external carotid artery (ECA) diameter (**a**) in a 69-year-old woman and (**b**) in a 76-year-old man (RadiAnt DICOM VIEWER, Medixant, Poland).

Preoperative and postoperative ECA diameters were measured in the pre- and postoperative angiograms at the narrowest point prior to the origin of the superior thyroid artery (Figure 1 and Figure 2).

Data on ICA stenosis, pre- and postoperative ECA diameters, and ECA stent coverage were collected from periprocedural angiograms using the RadiAnt DICOM VIEWER (Medixant, Poznań, Poland).

Pain occurrence and severity were recorded via a patient survey.

### 2.5. Statistical Analysis

The statistical analysis was performed using Statistica^®^ (Tulsa, OK, USA, 2013) software version 13.3 (StatSoft). Absolute values and percentages were used to present qualitative variables. Ranges, means, and standard deviations or medians with interquartile ranges were applied for quantitative variables. The Shapiro–Wilk test was used to determine statistical distribution in the analyzed patients. Between independent groups comparisons were performed using the chi-square test, Fisher’s exact test, or the Mann–Whitney U test. Comparison between dependent groups was performed with a Wilcoxon signed-rank test. A correlation analysis was performed using Spearman’s rank correlation coefficient. A *p*-value < 0.05 was considered statistically significant.

## 3. Results

### 3.1. Characteristics of the Study Group

The study group consisted of 55 adult patients (39, 70.9% men; 16, 29.1% women), aged 69 ± 7.53 SD (53–93).

Fifty patients (92.6%) had comorbidities. The most common was hypertension in 50 (92.6%), followed by dyslipidemia in 38 (70.4%), while 20 cases (37%) had diabetes. Forty-three (78.2%) patients showed manifestations of clinical symptoms before admission, which were associated with existing internal carotid artery stenosis. Dizziness was reported by 23 (41.8%) patients, headaches were reported by 11 patients (20%), and tinnitus was present in 5 (9.1%) patients. A history of stroke was found in 20 cases (37%). The characteristics of the study group are shown in Table 1.

### 3.2. Perioperative Data

The procedure was performed in 55 patients; in 39 (70.9%) cases, the procedure involved the left internal carotid artery, and in 16 (20.9%) patients, it involved the right carotid artery. The median stenosis value of the operated internal carotid artery was 80 IQR 24.5 (50–99%). All procedures were performed with distal neuroprotection. A stent was implanted in 55 cases; in 20 (37%) patients, the first-generation stent was used, while in 35 (63.6%) patients, the second-generation stent was used. In 54 (98.2%) patients, ECA patency was confirmed by angiography before stent insertion. After the procedure, the stent covered the ECA orifice in 53 (96.4%) patients. In 43 (78.2%) cases, there was a reduction in ECA diameter on follow-up angiographies; in six (10.9%), there was no change in vessel diameter; and in five (9.1%), there was a widening of ECA diameter. Additionally, in one (1.82%) patient, the post-procedural ECA occlusion was observed. The median ECA diameter on angiography before stent placement was 4.11 IQR 1.22 (0–6.84) mm, and after stent placement, it was 3.16 IQR 1.3 (0–5.97) mm. The difference between pre- and post-procedural ECA diameter was statistically significant (*p* < 0.001). The change in ECA diameter in mm is not dependent on the length and width of the stent used (*p* = 0.11, *p* = 0.28, respectively) as well as the stent type (*p* = 0.52) (Table 2).

**Figure 3:**
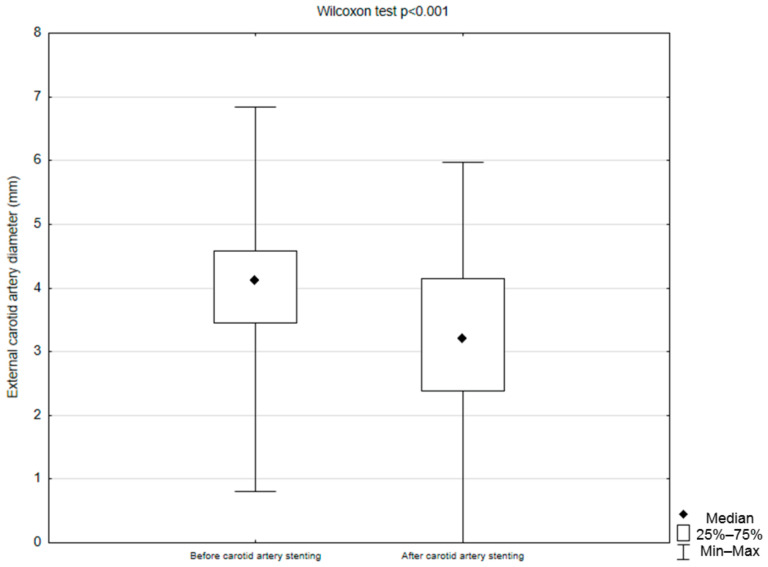
Comparison between external carotid artery diameter before and after carotid artery stenting (Statistica, StatSoft, USA).

There was a significant difference in pre- and postoperative ECA diameter (4.11 IQR 1.22 vs. 3.16 IQR 1.3 mm, *p* < 0.001) (Figure 3).

### 3.3. Perioperative Facial Pain

**Figure 4:**
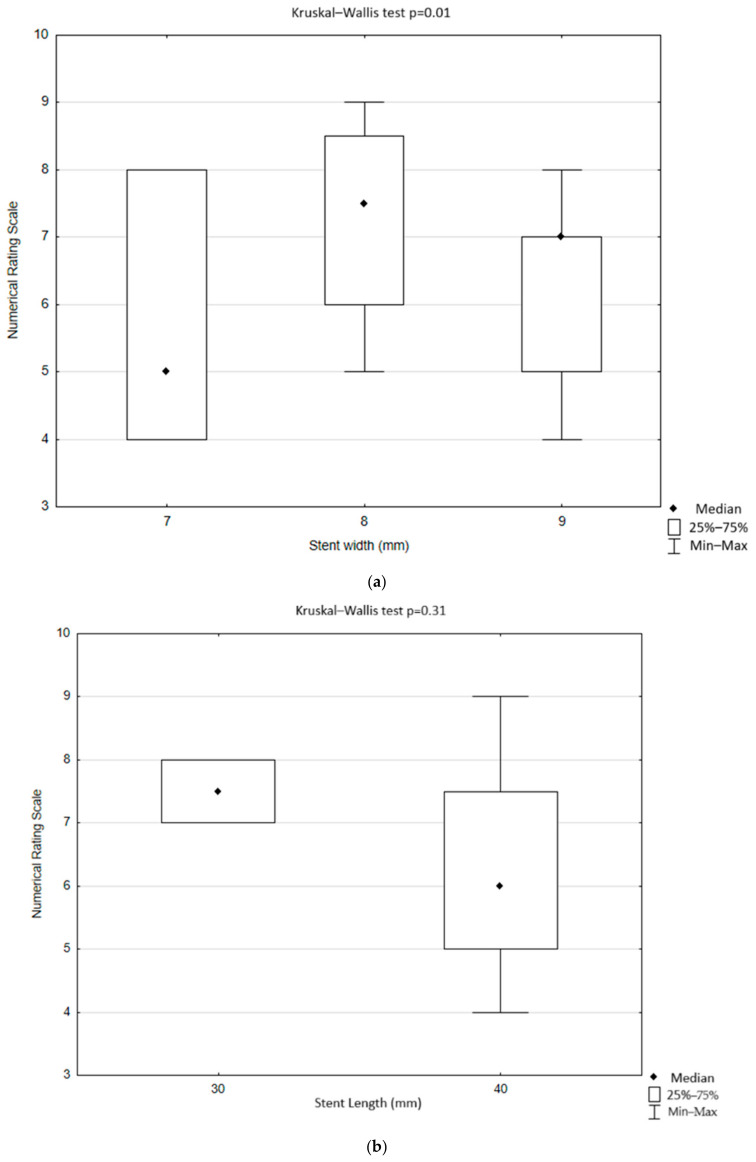
Facial pain severity during carotid artery stenting using the numerical rating scale depending on the (**a**) width of the carotid stent and (**b**) length of the carotid stent (Statistica, StatSoft, USA).

In the study group, most patients indicated a lack of pain during the stent expansion and subsequent balloon tightening (40; 72.73%). Fifteen (27.27%) patients reported pain during the procedure. The most indicated values of pain in the NRS were 5 and 7, and both were indicated by four (7.27%) patients, followed by the value of 8 on the NRS as indicated by three (5.45%) people. The presence of pain during the procedure did not depend on stent type (*p* = 0.27) nor on the width and length of the stent used (*p* = 0.15, *p* = 0.27, respectively) (Table 3 and Table 4). The severity of pain on the NRS did not depend on the type of the stent (*p* = 0.68) or the width and length of the stent used (*p* = 0.46, *p* = 0.31, respectively) (Figure 4).

**Figure 5:**
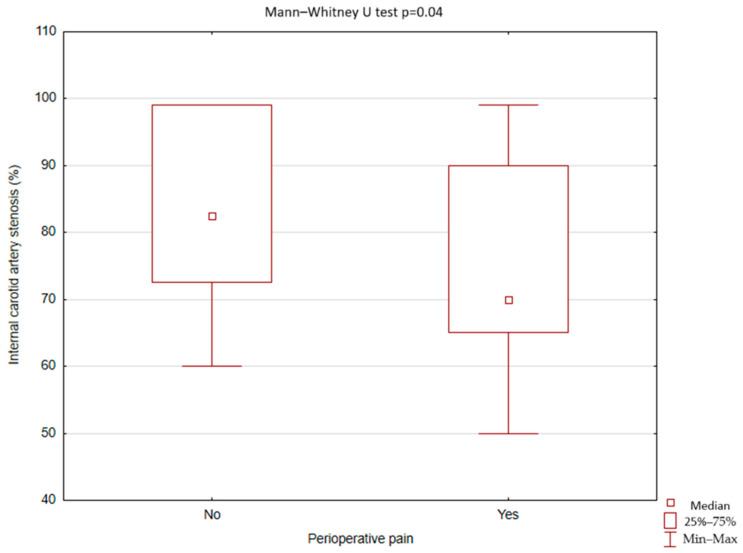
Occurrence of perioperative facial pain after carotid artery stenting depending on the internal carotid stenosis (Statistica, StatSoft, USA).

There was no statistically significant difference between patients with perioperative facial pain and patients without perioperative facial pain in terms of preoperative ECA diameter (4.23; 1.98–5,30 IQR 1.73 mm vs. 4.09; 0.8–6.84 IQR 1.07 mm, *p* = 0.82), postoperative ECA diameter (3.49; 0.9–4.4 IQR 2.19 mm vs. 3.1; 0–5.97 IQR 1.98 mm, *p* = 0.47), and in the difference between postoperative and preoperative ECA diameter (0.85; −0.6–2.19 IQR 0.95 mm vs. 0.71; −1.4–2.48 IQR1.33 mm, *p* = 0.57). Nonetheless, patients with perioperative facial pain had smaller ICA stenosis compared to the patients without perioperative facial pain (70; 50–99 IQR 25% vs. 82.5; 60–99 IQR 26.5%, *p* = 0.04) (Figure 5).

Additionally, no statistically significant correlation was found between perioperative facial pain severity and preoperative ECA diameter (R = 0.17, *p* = 0.57), postoperative ECA diameter (R = 0.04, *p* = 0.87), and the difference in postoperative and preoperative ECA diameter (R = 0.27, *p* = 0.38) as well as ICA stenosis (R = −0.06, *p* = 0.84).

### 3.4. Postoperative Facial Pain

Upon reexamination 24 h after the procedure, 51 (92.73%) patients reported no facial pain. On the other hand, other indicated values were 2, 3, 4, and 5 and each was indicated by one (1.82%) of the patients. There was no statistically significant difference in the occurrence of postoperative facial pain and the type of stent (*p* = 1) as well as stent width and length (*p* = 0.12, *p* = 0.87, respectively). There was also no statistically significant difference between postoperative facial pain severity and the type of stent (*p* = 0.68) as well as stent width and length (*p* = 0.65, *p* = 0.65, respectively) (Table 5 and Table 6).

**Figure 6:**
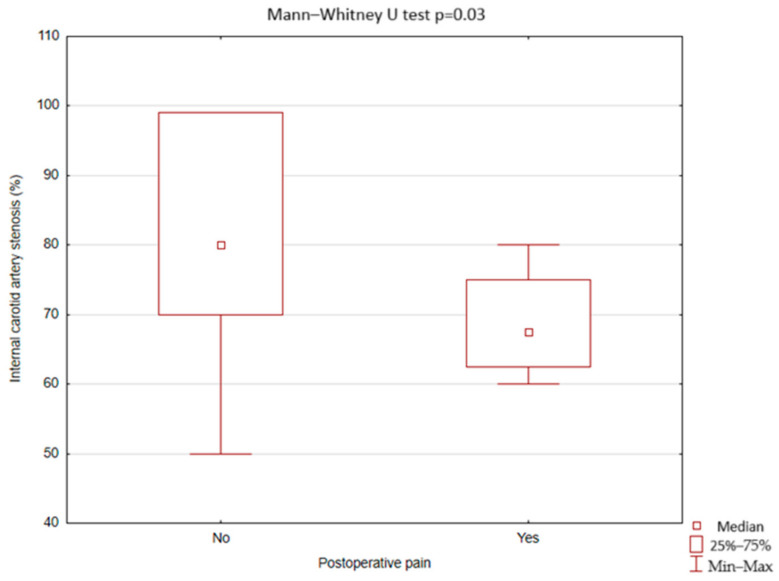
Occurrence of postoperative facial pain after carotid artery stenting depending on the internal carotid stenosis (Statistica, StatSoft, USA).

There was no statistically significant difference between patients with postoperative facial pain and patients without postoperative facial pain in terms of preoperative ECA diameter (4.61; 3–5.16 IQR 1.11 mm vs. 4.09; 0.8–6.84 IQR 1.05 mm, *p* = 0.31), postoperative ECA diameter (3.55; 2.39–3.9 IQR 0.8 mm vs. 3.13; 0–5.97 IQR 2.01 mm, *p* = 0.84), and in the difference between postoperative and preoperative ECA diameter (1.19; −0.6–2.19 IQR 1.86 mm vs. 0.7; −1.4–2.48 IQR 1.2 mm, *p* = 0.52). However, patients with perioperative facial pain had smaller ICA stenosis compared to the patients without perioperative facial pain (67.5; 60–80 IQR 12.5% vs. 80; 50–99 IQR 29%, *p* = 0.03) (Figure 6).

Additionally, no statistically significant correlation was found between perioperative facial pain severity and preoperative ECA diameter (R = 0.05, *p* = 1), postoperative ECA diameter (R = 0, *p* = 1), and the difference in postoperative and preoperative ECA diameter (R = −0.4, *p* = 0.6) as well as ICA stenosis (R = −0.04, *p* = 0.6).

## 4. Discussion

This study identified several important findings regarding the incidence and characteristics of facial pain associated with CAS. In 96.4% of patients, the stent placement resulted in coverage of the ECA orifice, a factor that was accompanied by a statistically significant reduction in the ECA diameter. Specifically, the median preoperative ECA diameter of 4.11 mm decreased to 3.16 mm following the procedure, reflecting a considerable change in vessel diameter. Among the 55 patients, the majority (72.73%) did not experience facial pain during CAS, while 27.27% reported varying degrees of pain. The occurrence and intensity of pain were not significantly correlated with the length or width of the stent used. However, a relationship was observed between the degree of ICA stenosis and the presence of perioperative facial pain, with patients exhibiting lower ICA stenosis being more likely to report pain during the procedure. Patients’ evaluations 24 h after the procedure showed that 92.73% of patients were free from facial pain, with only a few reporting mild discomfort. Nonetheless, postoperative assessments indicated that patients with lower ICA stenosis were also more likely to experience facial pain following the procedure.

The ECA is considered a potential contributor to collateral blood supply to the brain. Many specialists suggest that the ECA serves as a critical source of ipsilateral cerebral blood flow, especially in cases of ICA stenosis or occlusion [11]. Studies indicate that with increasing ICA stenosis, ECA contribution to middle cerebral artery (MCA) blood flow may reach up to 15% of total MCA flow [24,25]. Conversely, some researchers question the significance of ECA in cerebral perfusion, suggesting that its role in maintaining adequate brain blood flow remains uncertain [26]. Therefore, the ECA’s fate cannot be underestimated.

In our study, overstenting—defined as the coverage of the ECA orifice by a stent—was observed in 52 patients (96.4%). To our knowledge, the only previous study to report on the incidence of ECA overstenting was conducted by de Borst et al. [11], where overstenting occurred in 75% of cases. These findings indicate that ECA overstenting is a common occurrence during CAS procedures. Given the ECA’s role in potential collateral cerebral perfusion, particularly in cases of ICA stenosis, these results underscore the importance of monitoring the ECA as carefully as the ICA post-CAS. Continued assessment of ECA patency and function may be crucial for understanding the broader hemodynamic impacts of CAS and for managing potential complications associated with reduced ECA flow.

In our cohort, a reduction in ECA diameter following CAS was observed in 43 patients (78.2%). The change in ECA diameter post-procedure was statistically significant, with a median preoperative measurement of 4.11 mm (IQR 1.22; range 0–6.84 mm) compared to a postoperative median of 3.16 mm (IQR 1.3; range 0–5.97 mm). To the best of our knowledge, no previous studies have documented pre- and post-procedural ECA diameters using angiographic measurements. However, de Borst et al. [20] reported a progressive increase in the incidence of ≥50% ECA stenosis, from 29.9% at 3 months to 65.8% at 60 months post-CAS with an overstented ECA. Additionally, the study found that stenosis progression was greater in overstented ECAs compared to the contralateral side. Similarly, Willfort-Ehringer et al. [27] noted an increase in ≥70% ECA stenosis from 19.29% at 3 months to 38.32% at 24 months post-CAS in cases of ECA overstenting. This stenosis progression may be attributable to displacement of atheromatous material from the CCA/ICA into the ECA orifice during CAS, as well as to flow disturbances caused by blood passing through the stent mesh, potentially exacerbating ECA narrowing over time.

Our study observed that CAS was associated with facial pain in 27.27% of patients during the procedure, with 7.28% reporting residual facial pain 24 h post-procedure. To our knowledge, this is the first study specifically reporting the occurrence of facial pain in patients undergoing CAS. Furthermore, both peri- and postoperative facial pain were more prevalent among patients with lower degrees of ICA stenosis. In their study, Shirley et al. [28] investigated the potential correlation between the severity of ICA stenosis and ECA to cerebral blood flow. After clamping the ECA, they observed a median decrease in cerebral blood flow measured by transcranial Doppler sonography of 13.2% in patients with >90% ICA stenosis and 11.5% in those with <90% stenosis. However, these findings were not statistically significant. Additionally, Kaszczewski et al. [28,29] demonstrated that the number of patients with increased blood flow volume in both the ECA and vertebral arteries rises with the degree of ICA stenosis, with the volumetric flow compensation predominantly occurring in the ECA.

Moreover, the authors of this article suggest that the observed finding of a higher occurrence of facial pain in a lower percentage of ICA stenosis may be linked to the limited role of the ECA in cerebral blood flow in cases of lower degrees of ICA stenosis. This limitation may lead to insufficient or absent collateral supply to the facial region. Consequently, impaired blood flow in the ECA following CAS may result in facial pain due to inadequate collateral blood supply to the face.

Previous studies have documented other clinical symptoms related to reduced ECA blood flow following CAS. For instance, Giurgea et al. [30] reported cases of jaw claudication post-CAS, with time to onset nearly halving post-procedure (90 s) compared to pre-procedure (190 s). Although recovery time improved to 150 s within a week post-CAS, it did not return to baseline levels. Additionally, other studies have reported acute hemifacial ischemia as a long-term complication following CAS [31]. Moreover, in the study by Kim et al. [32], the presence of atherosclerotic plaque causing ECA stenosis emerged as an independent predictor of all-cause mortality, with a hazard ratio of 2.6. These findings highlight the importance of monitoring the ECA’s role in maintaining adequate facial and cerebral perfusion following CAS, especially given the observed incidence of facial pain, jaw claudication, and hemifacial ischemia. The association of facial pain with lower ICA stenosis suggests that ECA flow alterations during CAS may have a direct impact on facial vascular supply, particularly when the ECA’s compensatory role is less pronounced. Additionally, the correlation between ECA stenosis and increased mortality, as shown in Kim et al. [32], underscores the need to carefully assess and monitor ECA patency in CAS patients. Together, these observations indicate that while CAS effectively addresses ICA stenosis, its effects on the ECA should not be overlooked, as they may contribute to both immediate symptoms and longer-term risks.

### Limitations of the Study

Our study has several limitations. First, the pain reported by patients is subjective and may differ between individuals due to varying pain tolerance levels. Although the numeric rating scale (NRS) is widely used for its simplicity and ease of application, it has notable limitations. The scale relies heavily on patient self-report, which can be influenced by individual variability in pain perception, emotional state, or cultural background. Furthermore, the NRS measures only the intensity of pain, disregarding other important dimensions such as pain quality, duration, or the psychological and functional impact of pain. Second, the lack of blood flow measurements limits conclusions about hemodynamic changes in the ECA after CAS. Third, the absence of long-term follow-up restricts data on long-term outcomes and complications associated with ECA overstenting. Finally, the single-center design may limit the generalizability of our findings to a broader population.

## 5. Conclusions

In most cases, CAS results in overstenting of the ECA, leading to a significant reduction in its diameter. This effect can be associated with the occurrence of perioperative facial pain, especially in patients with lower degrees of ICA stenosis. Although most patients experienced a decrease in facial pain within 24 h, the initial incidence of facial pain highlights a need for careful consideration of ECA patency in CAS procedures.

## Figures and Tables

**Table 1:** Study group characteristics.

Variable	*n* (%), Mean/Median (Range, SD/IQR)
Age (years)	69 (53–93, SD 7.53)
Gender	
Male	39 (70.9%)
Female	16 (29.1%)
Current cigarette smoking	22 (40.7%)
Presence of comorbidities (yes)	50 (92.6%)
Arterial hypertension	50 (92.6%)
Dyslipidemia	38 (70.4%)
Diabetes mellitus	20 (37%)
Clinical symptoms (yes)	43 (78.2%)
Dizziness	23 (41.8%
Stroke	20 (37%)
Headache	11 (20%)
Syncope	6 (10.9%)
Tinnitus	5 (9.1%)

Abbreviation: SD—standard deviation; IQR—interquartile range.

**Table 2:** Perioperative data.

Variable	*n* (%), Mean/Median (Range, SD/IQR)
ICA stenosis (%)	80 (50–99) IQR 24.5
Side of the procedure	
Left ICA	39 (70.9%)
Right ICA	16 (29.1%)
Stent used	
1st generation	20 (37%)
2nd generation	35 (63.6%)
Stent length	
40 mm	39 (70.9%)
30 mm	15 (27.3%)
60 mm	1 (1.8%)
Stent width	
8 mm	22 (40%)
9 mm	15 (27.3%)
7 mm	12 (21.8%)
10 mm	6 (10.9%)
Covering of ECA orifice by a carotid stent	
Yes	53 (96.4%)
No	2 (3.6%)
ECA diameter before CAS (mm)	4.11 (0–6.84) IQR 1.22
ECA diameter after CAS (mm)	3.16 (0–5.97) IQR 1.78

Abbreviations: SD—standard deviation; IQR—interquartile range; CAS—carotid artery stenting; ECA—external carotid artery; ICA—internal carotid artery.

**Table 3:** Presence of facial pain during the procedure depending on the stent width.

Presence of Pain During the Procedure	Stent Width 7 mm *n* = 12	Stent Width 8 mm *n* = 22	Stent Width 9 mm *n* = 15	Stent Width 10 mm *n* = 6	Total *n* = 55	*p*
No	9 (75%)	17 (77.17%)	8 (53.33%)	6 (100%)	40 (72.73%)	0.15
Yes	3 (25%)	5 (22.73%)	7 (46.67%)	0 (0%)	15 (27.27%)	

**Table 4:** Presence of facial pain during the procedure depending on the stent length.

Presence of Pain During the Procedure	Stent Length 30 mm *n* = 15	Stent Width 40 mm *n* = 39	Stent Width 60 mm *n* = 1	Total *n* = 55	*p*
No	13 (86.67%)	26 (66.67%)	1 (100%)	40 (72.73%)	0.27
Yes	2 (13.33%)	13 (33.33%)	0 (0%)	15 (27.27%)	

**Table 5:** Presence of facial pain 24 h after the procedure depending on the stent width.

Presence of Pain During the Procedure	Stent Width 7 mm *n* = 12	Stent Width 8 mm *n* = 22	Stent Width 9 mm *n* = 15	Stent Width 10 mm *n* = 6	Total *n* = 55	*p*
No	12 (91.67%)	22 (100%)	12 (80%)	6 (100%)	51 (92.73%)	0.12
Yes	1 (8.33%)	0 (0%)	3 (20%)	0 (0%)	4 (7.27%)	

**Table 6:** Presence of facial pain 24 h after the procedure depending on the stent length.

Presence of Pain During the Procedure	Stent Length 30 mm *n* = 15	Stent Width 40 mm *n* = 39	Stent Width 60 mm *n* = 1	Total *n* = 55	*p*
No	14 (93.33%)	36 (92.31%)	1 (100%)	51 (92.73%)	0.87
Yes	1 (6.67%)	3 (7.69%)	0 (0%)	4 (7.27%)	

## Data Availability

The original contributions presented in this study are included in the Supplementary Materials. Further inquiries can be directed to the corresponding authors.

## Supplementary Materials

The following supporting information can be downloaded at https://www.mdpi.com/article/10.3390/jcm13247666/s1: Supplementary Materials—Raw data set of the study.
