# Supplementary material for: Assessment of Facial Pain After Internal Carotid Artery Stenting: The Role of External Carotid Artery Overstenting
Source: J Clin Med. 2024 Dec 16;13(24):7666. doi: 10.3390/jcm13247666 (PMC11676484; doi:10.3390/jcm13247666)
Supplement: Supplementary file 1 [file jcm-13-07666-s001.zip › jcm-3329176-supplementary.pdf]

Płeć	Wiek	Używki tak= 1 nie=0 brak danych = 2	Papierosy tak= 1 nie=0	Alkohol tak= 1 nie=0	Choroby towarzyszące tak= 1 nie= 0	Nadciśnienie tętnicze tak= 1 nie=0
M	69	0	0	0	0	0
M	85	0	0	0	1	1
M	77	0	0	0	1	1
M	64	0	0	0	1	1
M	64	1	1	0	1	1
K	71	0	0	0	1	1
M	73	1	1	0	1	1
M	72	1	1	0	1	1
M	70	1	1	0	1	1
M	66	0	0	0	1	1
M	68	0	0	0	1	1
K	68	1	1	0	1	1
M	66	1	1	0	1	1
K	57	1	1	0	1	1
K	76	1	1	0	1	1
K	71	0	0	0	1	1
M	66	1	1	0	1	1
K	71	1	1	0	1	1
M	63	0	0	0	1	1
M	79	1	1	0	1	1
M	65	1	1	0	1	1
M	67	1	1	0	0	0
M	66	1	1	0	1	1
K	58	0	0	0	1	1
M	64	0	0	0	1	1
M	88	0	0	0	1	1
K	65	0	0	0	1	1
M	70	0	0	0	1	1
K	65	0	0	0	0	0
K	81	0	0	0	1	1
M	76	0	0	0	1	1
K	62	0	0	0	1	1
M	74	1	1	0	1	1
K	72	1	1	0	1	1

K	93	0	0	0	1	1
M	80	0	0	0	1	1
M	62	0	0	0	1	1
M	53	0	0	0	1	1
M	69	0	0	0	0	0
K	66	0	0	0	1	1
K	66	0	0	0	1	1
M	81					
M	59	0	0	0	1	1
M	65	0	0	0	1	1
M	68	0	0	0	1	1
M	79	0	0	0	1	1
M	70	0	0	0	1	1
M	72	1	1	1	1	1
K	70	0	0	0	1	1
M	69	1	1	0	1	1
M	69	1	1	0	1	1
M	72	1	1	1	1	1
M	72	0	0	0	1	1
M	72	1	1	1	1	1
M	79	1	1	1	1	1

Dyslipidemia tak= 1 nie=0	Cukrzyca tak= 1 nie=0	objawy kliniczne tak=1 brak=0	Omdlenia 1 = tak 0=nie	ból głowy tak= 1 nie=0	zawroty głowy tak= 1 nie=0	szumy uszne tak= 1 nie=0
0	0	1	0	0	0	0
1	1	0	0	0	0	0
1	0	0	0	0	0	0
1	0	0	0	0	0	0
1	0	1	0	0	0	1
1	0	1	1	1	1	0
1	1	1	0	1	1	0
1	0	1	0	1	0	0
1	0	1	0	1	0	0
1	1	1	0	0	0	0
1	0	1	0	1	1	0
1	0	0	0	0	0	0
1	0	1	0	0	1	0
1	0	1	0	0	0	0
1	0	1	0	0	1	0
1	1	1	0	0	1	0
1	0	1	0	1	1	1
1	0	0	0	0	1	1
1	1	1	0	0	1	0
1	1	0	0	0	0	0
0	0	1	0	0	0	0
0	0	1	0	0	0	0
0	0	1	0	0	0	0
1	0	1	0	0	0	0
0	0	1	0	0	0	0
0	0	1	0	0	1	0
1	1	1	1	0	1	0
1	0	1	0	1	1	0
0	0	1	0	0	0	0
0	0	0	0	0	0	0
1	1	1	0	0	0	0
1	1	0	0	0	0	0
1	1	1	0	0	1	0
1	0	1	0	0	1	0

1	0	1	1	1	1	0
0	0	1	0	0	0	0
1	1	1	0	0	0	0
1	0	1	0	0	0	0
0	0	0	0	0	0	0
1	0	0	0	0	0	0
1	1	0	0	0	0	0
		0	0	0	0	0
0	1	1	0	0	0	0
0	0	1	0	0	1	0
1	1	1	0	1	0	0
0	0	1	0	1	1	0
1	1	1	1	0	0	0
1	1	1	0	0	0	0
1	1	1	1	0	0	1
1	1	1	0	0	1	0
0	0	1	0	0	1	0
1	1	1	1	0	1	0
0	0	1	0	0	1	0
1	0	1	0	0	1	1
1	1	1	0	1	1	0

udar tak= 1 nie=0	TIA tak= 1 nie=0	Inne objawy tak=1 nie=0	Jakieobjawy	LICA drożna tak - 1 nie-% stenozy	LECA drożna tak - 1 nie- % stenozy	RICA drożna tak - 1 nie- % stenozy
1	0	0	0	95	1	70
0	0	0	0	80	1	90
0	0	0	0	80	1	1
0	0	0	0	80	1	1
1	0	0	osłabienie	99		50
0	0	0		70	1	1
0	0	0	0	99	1	80
1	0	0	0	70	1	1
0	0	0	ból lewego oka rano	1	1	80
1	0	0	0	1	1	80
1	0	0	0	60	1	40
0	0	0	0	1	1	1
0	0	0	0	90	1	1
0	0	0	0	99	1	80
0	0	0	0	80	1	1
0	0	0	zaburzenia równowagi	1	1	99
0	0	0	0	1	1	1
0	0	0	zaburzenia orientacji przestrzennej i zaburzenia widzenia	90	1	1
0	0	0	0	60	1	90
0	0	0	0	90	1	1
1	0	0	0	70	1	1
1	0	0	0	1	1	60
1	0	0	0	95		
0	1	0	0	70	1	70
1	0	0	0	1	1	75
0	0	0	0	70	1	1
0	0	0	0	90	1	90
0	0	0	0	70	1	80
1	0	0	0	100	1	70
0	0	0	0	1	1	70
1	0	0	0	99	1	1
0	0	0	0	90	1	1
1	0	0	0	70	1	40
0	0	0	drętwienie lewej kończyny górnej	90	1	1

0	0	0	brak czucia w kciuki lewym, bóle kończyny górnej lewej	70	1	1
1	0	0	zaburzenia czucia języka,	90	1	1
0	0	0	zaburzenia widzenia (przymglenie obrazu)	90	1	1
0	1	0	zaburzenia widzenia (falowanie obrazu), problemy z koncentracją,	70	1	90
0	0	0		90	1	65
0	0	0		80	1	100
0	0	0		90	1	1
0	0	0		75	1	1
0	0	0		65	1	100
1	0	0	problemy z koncentracją	80	1	1
1	0	0	napadowe bóle głowy o umiarkowanym natężeniu ok 6/10	90	1	80
0	0	0	ból skroni obustronny	50	1	100
0	0	0	ból ramion i rąk 8/10	80	1	85
0	0	0	podwójne widzenie	90	1	1
1	0	0	niepokój, nagłe wymioty	1	1	90
0	0	0	mroczki przed oczami	1	80	1
0	0	0	napadowe zamglone	1	1	80
1	0	0	0	100	1	80
0	0	0	0	90	1	1
0	0	0	0	50	1	1
0	0	0	0	70	1	1

RECA drożna tak - 1 nie- % stenozy	Skala NIHSS przy przyjęciu	Predylatacja balonem 1- tak 0- nie	Neuroprotekcja 1- Spider FX 2-Emboshield 3- EPI FILTER 4- FIlterwire 5- Proender 6- MoMa	rodzaj stentu 1- Precise PRO RX 2- CGuard Carotid EPS 3 -Wallstent	Szerokość stentu (mm)	Długość (mm)
1	0	0	1	2	7	40
1	0	0	1	1	8	40
1	0	0	1	2	9	30
1	0	0	1	1	8	40
1	1	1	1	2	7	40
1	0	0	3	3	9	30
1	0	1	4	2	7	40
	0	0	1	1	8	40
1	0	1	4	2	10	60
1	0	0	1	2	9	40
1	3	1	3	2	9	40
1	0	0	4	2	9	40
1	0	1	1	1	8	40
1	0	1	1	1	7	30
1	0	0	1	1	7	30
1	0	0	1	2	8	30
70	0	1	1	2	9	40
1	0	1	1	2	8	40
1	0	0	4	1	8	40
1	0	1	3	2	7	40
1	0	0	3	2	9	40
1	3	0	3	3	9	30
	2	0	1	2	8	40
1	0	1	1	3	7	40
1	9	1	1	2	8	40
1	0	0	4	1	8	30
1	0	1	1	1	8	30
1	0	1	1	1	8	30
1	6	1	1	2	9	40
1	0	1	1	2	9	40
1	1	1	2	2	7	30
1	0	1	2	2	8	40
1	0	0	1	2	7	30
1	0	0	4	1	8	40

1	0	1	2	1	7	30
1	0	0	3	2	8	30
1	0	1	1	2	7	40
1	0	1	3	2	8	40
1	0	1	1	2	10	40
1	0	1	3	1	8	40
1	0	1	5	2	8	40
1	0	1	2	2	10	40
1	0	1	3	2	9	40
1	0	1	2	2	10	40
1	0	0	2	2	8	40
1	0	1	2	2	9	40
1	0	1	1	2	8	40
1	0	0	1	2	8	30
1	0	0	3	1	7	30
1	0	0	4	1	9	40
1	0	0	4	1	9	40
1	1	0	1	1	10	40
1	0	0	5	2	9	40
90	0	0	4	2	10	40
1	0	0	4	2	8	40

Strona do zabiegu 1-prawa 2-lewa	Czas trwania zabiegu	Zabieg skuteczny tak=1 nie=0 konwersja do endarterektomi =2	Przyczyna	% Stenozy operowanej ICA (w wolnym czasie przejrzeć i policzyć na nowo zgodnie ze wzorem NASCET A-B/A		Subokluzja 0-nie 1-tak
2	20	1		90.00	90.00	0
2	40	1		80.00	80.00	0
2	45	1		80.00	80.00	0
2	30	1		80.00	80.00	0
2	45	1		99.00	99.00	1
2	20	1		70.00	70.00	0
2	40	1		99.00	99.00	1
2	25	1		70.00	70.00	0
1	40	1		80.00	80.00	0
1	35	1		80.00	80.00	0
2	65	1		60.00	60.00	0
2	20	1		90.00	90.00	0
2	25	1		90.00	90.00	0
2	35	1		99.00	99.00	1
2	35	1		80.00	80.00	0
1	25	1		99.00	99.00	1
1	20	1		70.00	70.00	0
2	35	1		99.00	99.00	1
1	25	1		90.00	90.00	0
2	35	1		90.00	90.00	0
2	20	1		70.00	70.00	0
1	20	1		60.00	60.00	0
2	45	1		99.00	99.00	1
2	35	1		70.00	70.00	0
1	30	1		65.00	65.00	1
2	30	1		70.00	70.00	0
2	30	1		99.00	99.00	1
1	30	1		80.00	80.00	0
1	60	1		70.00	70.00	1
1	60	1		70.00	70.00	0
2	60	1		99.00	99.00	1
2	60	1		90.00	90.00	0
2	90	1		70.00	70.00	0
2	60	1		60.00	60.00	0

2	75	1	70.00	70.00	0
2	25	1	99.00	99.00	1
2	40	1	99.00	99.00	1
1	40	1	99.00	99.00	1
2	40	1	90.00	90.00	0
2	60	1	80.00	80.00	0
2	45	1	99.00	99.00	1
2	70	1	90.00	90.00	0
2	25	1	90.00	90.00	0
2	45	1	80.00	80.00	0
1	20	1	90.00	90.00	0
2	35	1	90.00	90.00	0
1	25	1	85.00	85.00	0
2	25	1	99.00	99.00	1
2	35	1	99.00	99.00	1
2	35	1	80.00	80.00	0
1	40	1	70.00	70.00	0
1	60	1	80.00	80.00	0
2	40	1	90.00	90.00	0
1	25	1	90.00	90.00	0
2	40	1	70.00	70.00	0

Full colaps(przepły w strunowaty w całym naczyniu) tak=1 nie=0	Średnica ICA przed zabiegiem mm	Średnica ICA po zabiegu (mm)	Różnica w średnicy po zabiegu (mm)	Zmiana średnicy ICA po zabiegu 0= brak zmiany 1= zwiększenie 2= zmiejszenie	Drożna ECA przed zabiegiem tak = 1 nie = 0	Średnica ECA przed zabiegiem mm
0	2.52	4.63	2.11	1	1	3.6
0	2.47	5.27	2.8	1	1	4.00
0	2.03	5.38	3.35	1	1	5.30
0	2.14	4.06	1.92	1	1	4.40
0	1.08	5.13	4.05	1	1	4.40
0	1.73	4.83	3.1	1	1	4.20
0	1.4	3.92	2.52	1	1	3.60
0	2.15	4.25	2.1	1	1	4.20
0	1.45	6.73	5.28	1	1	4.70
0	4.92	6.04	1.12	1	1	4.50
0	3.45	5.42	1.97	1	1	3.00
0	4.51	5.55	1.04	1	0	0.00
0	2.18	4.93	2.75	1	1	5.10
0	0.78	5	4.22	1	1	3.50
0	2.28	4.99	2.71	1	1	3.00
0	1.4	4.7	3.3	1	1	3.60
0	2.9	7.13	4.23	1	1	5.30
0	0.63	4.9	4.27	1	1	2.00
0	2.45	5.35	2.88	1	1	4.80
0	2.47	4.03	1.56	1	1	3.50
	3.58	5.87	2.29	1	1	3.00
	1.87	4.32	2.45	1	1	2.90
0	0.87	3.55	2.68	1	1	4.00
	1.02	3.69	2.67	1	1	2.85
0	0.9	5.4	4.5	1	1	4.25
	2.54	5.37	2.83	1	1	3.00
0	1.12	4.66	3.54	1	1	3.45
	2.17	5.28	3.11	1	1	4.62
0	1.05	5.33	4.28	1	1	3.70
	2.32	4.68	2.36	1	1	5.70
0	0.8	6.7	5.9	1	1	4.07
0	1.2	3.81	2.61	1	1	3.20
0	3.02	5.38	2.36	1	1	4.58
0	3.5	5.44	1.94	1	1	4.13

0	5.08	6.91	1.83	1	1	3.11
0	1.53	5.98	4.45	1	1	4.95
0	1	5.66	4.66	1	1	3.72
0	1.22	4.95	3.73	1	1	4.35
0	2.34	6.51	4.17	1	1	5.2
0	2.95	5.11	2.16	1	1	2.52
0	1	4	3	1	1	0.8
0	5.29	5.29	0	0	1	4.33
0	1.36	3.68	2.32	1	1	5.16
0	1.94	3.6	1.66	1	1	4.38
0	3.35	4.91	1.56	1	1	1.98
0	1.45	3.88	2.43	1	1	4.51
0	2.34	6.21	3.87	1	1	4.39
0	0.68	5.11	4.43	1	1	2.3
0	1.2	3.91	2.71	1	1	3.47
0	3.46	6.25	2.79	1	1	4.63
0	3.3	7.63	4.33	1	1	3.91
0	5.16	6.55	1.39	1	1	6.84
0	1.72	5.65	3.93	1	1	5.38
0	4.61	5.13	0.52	1	1	6.8
0	2.6	5.13	2.53	1	1	5.6

Średnica ECA po zabiegu (mm)	Różnica w średnicy ECA po zabiegu (mm)	Zmiana średnicy ECA po zabiegu 0= brak zmiany 2= zwiększenie 1= zmiejszenie	Czy stent przykrywa ECA tak =1 nie = 0	Powikłania w trakcie zabiegu tak=1 nie=0	Omdlenie tak=1 nie=0	Udar tak=1 nie=0
2.25	1.35	1	1	0	0	0
3.40	0.60	1	1	0	0	0
5.30	0.00	0	1	0	0	0
4.40	0.00	0	1	0	0	0
3.10	1.30	1	1	0	0	0
3.90	0.30	1	1	0	0	0
1.85	1.75	1	1	0	0	0
2.32	1.88	1	1	0	0	0
4.70	0.00	0	1	0	0	0
3.90	0.60	1	1	0	0	0
3.60	-0.60	2	1	0	0	0
0.00	0.00	0	0	0	0	0
4.40	0.70	1	1	0	0	0
3.10	0.40	1	1	0	0	0
4.40	-1.40	2	1	0	0	0
3.50	0.10	1	1	0	0	0
3.90	1.40	1	1	0	0	0
0.90	1.10	1	1	0	0	0
4.80	0.00	0	1	0	0	0
2.60	0.90	1	1	0	0	0
3.00	0.00	0	1	0	0	0
2.50	0.40	1	1	0	0	0
2.60	1.40	1	1	0	0	0
1.60	1.25	1	1	0	0	0
3.25	1.00	1	1	0	0	0
2.25	0.75	1	1	0	0	0
2.30	1.15	1	1	0	0	0
2.14	2.48	1	1	0	0	0
1.79	1.91	1	1	0	0	0
4.80	0.90	1	1	0	0	0
4.33	-0.26	2	0	0	0	0
0.84	2.36	1	1	0	0	0
2.39	2.19	1	1	0	0	0
4.13	0	0	1	0	0	0

2.43	0.68	1	1	0	0	0
3.57	1.38	1	1	0	0	0
3.48	0.24	1	1	0	0	0
2.96	1.39	1	1	0	0	0
5.12	0.08	1	1	0	0	0
1.3	1.22	1	1	0	0	0
0	0.8	1	1	0	0	0
4.44	-0.11	2	1	0	0	0
3.51	1.65	1	1	0	0	0
4.15	0.23	1	1	0	0	0
1.71	0.27	1	1	0	0	0
3.92	0.59	1	1	0	0	0
2.98	1.41	1	1	0	0	0
2.42	-0.12	2	1	0	0	0
3.16	0.31	1	1	0	0	0
3.9	0.73	1	1	0	0	0
2.6	1.31	1	1	0	0	0
5.97	0.87	1	1	0	0	0
4.68	0.7	1	1	0	0	0
4.4	2.4	1	1	0	0	0
2.2	3.4	1	1	0	0	0

Zgon tak=1 nie=0	Przyczyna	Inne jakie	Skala NIHSS 24 h po zabiegu	Badanie neurologiczne tuż po zabiegu świeże ubytki neurologiczne tak-1 nie-0	Jakie ubytki?	Badanie neurologiczne 24h po zabiegu 0-prawidłowe 1-świeże ubytki neurologiczne
0	0	0	0	0	0	0
0	0	0	0	0	0	0
0	0	0	0	0	0	0
0	0	0	0	0	0	0
0	0	0	1	0	0	0
0	0	0	0	0	0	0
0	0	0	0	0	0	0
0	0	0	0	0	0	0
0	0	0	0	0	0	0
0	0	0	0	0	0	0
0	0	0	3	0	0	0
0	0	0	0	0	0	0
0	0	0	0	0	0	0
0	0	0	0	0	0	0
0	0	0	0	0	0	0
0	0	0	0	0	0	0
0	0	0	0	0	0	0
0	0	0	0	0	0	0
0	0	0	0	0	0	0
0	0	0	0	0	0	0
0	0	0	0	0	0	0
0	0	0	3	0	0	0
0	0	0	2	0	0	0
0	0	0	0	0	0	0
0	0	0	9	0	0	0
0	0	0	0	0	0	0
0	0	0	0	0	0	0
0	0	0	0	0	0	0
0	0	0	6	0	0	0
0	0	0	0	0	0	0
0	0	0	1	0	0	0
0	0	0	0	0	0	0
0	0	0	0	0	0	0
0	0	0	0	0	0	0

0	0	0	0	0	0	0
0	0	0	0	0	0	0
0	0	0	0	0	0	0
0	0	0	0	0	0	0
0	0	0	0	0	0	0
0	0	0	0	0	0	0
0	0	0	0	0	0	0
0	0	0	0	0	0	0
0	0	0	0	0	0	0
0	0	0	0	0	0	0
0	0	0	0	0	0	0
0	0	0	0	0	0	0
0	0	0	0	0	0	0
0	0	0	0	0	0	0
0	0	0	0	0	0	0
0	0	0	0	0	0	0
0	0	0	0	0	0	0
0	0	0	1	0	0	0
0	0	0	0	0	0	0
0	0	0	0	0	0	0
0	0	0	0	0	0	0

Restenoza ICA	Przemieszczenie stentu 1 = tak 0=nie	Inne powikłamia	Skala NIHSS przy wypisie	Czas pobytu w szpitalu (dni)	Czas pobytu po zabiegu (dni)	Powikłania wczesne do 30 po zabiegu 1= tak 0=nie
0	0	0	0	2	1	0
0	0	0	0	2	1	0
0	0	0	0	3	2	0
0	0	0	0	3	2	0
0	0	0	1	2	1	0
0	0	0	0	3	2	0
0	0	0	0	2	1	0
0	0	0	0	2	1	0
0	0	0	0	3	2	0
0	0	0	0	3	1	0
0	0	0	3	5	1	0
0	0	0	0	3	2	0
0	0	0	0	2	1	0
0	0	0	0	4	1	0
0	0	0	0	3	2	0
0	0	0	0	3	2	0
0	0	0	0	2	1	0
0	0	0	0	2	1	0
0	0	0	0	2	1	0
0	0	0	0	2	1	0
0	0	0	0	3	1	0
0	0	0	3	2	1	0
0	0	0	2	2	1	0
0	0	0	0	3	2	0
0	0	0	9	3	2	0
0	0	0	0	3	2	0
0	0	0	0	3	2	0
0	0	0	0	2	1	0
0	0	0	6	3	2	0
0	0	0	0	3	2	0
0	0	0	1	3	2	0
0	0	0	0	3	2	0
0	0	0	0	4	2	0
0	0	0	0	2	1	0

0	0	0		2	1	0
			0			
0	0	0	0	2	1	0
0	0	0		3	2	0
			0			
0	0	0		2	1	0
			0			
0	0	0	0	4	2	0
0	0	0	0	2	1	0
0	0	0	0	3	2	0
0	0	0	0	3	2	0
0	0	0	0	3	2	0
0	0	0	0	2	1	0
0	0	0		12	11	1
			0			
0	0	0	0	2	1	0
0	0	0	0	2	1	0
0	0	0	0	2	1	0
0	0	0	0	2	1	0
0	0	0	0	2	1	0
0	0	0	0	2	1	0
0	0	0	1	2	1	0
0	0	0	0	3	2	0
0	0	0	0	2	2	0
0	0	0	0	4	1	0

Czas od zabiegu (dni)	udar 1 = tak 0=nie	Tia 1= tak 0 = nie	zawał mięśnia sercowego 1 = tak 0=nie	zespół hipoperfuzji 1 = tak 0=nie	inne powikłanmia 1- jatrogenne uszkodzenie tętnicy żołądkowej lewej i wstrząs krowotoczny 2- krwiak zaotrzewnowy 3 -Zaostrzenie POCHP 4-	Zgon 1 = tak 0=nie
	0	0	0	0	0	0
	0	0	0	0	0	0
	0	0	0	0	0	0
	0	0	0	0	0	0
	0	0	0	0	0	0
	0	0	0	0	0	0
	0	0	0	0	0	0
	0	0	0	0	0	0
	0	0	0	0	0	0
	0	0	0	0	0	0
	0	0	0	0	0	0
	0	0	0	0	0	0
	0	0	0	0	0	0
	0	0	0	0	0	0
	0	0	0	0	0	0
	0	0	0	0	0	0
	0	0	0	0	0	0
	0	0	0	0	0	0
	0	0	0	0	0	0
	0	0	0	0	0	0
	0	0	0	0	0	0
	0	0	0	0	0	0
	0	0	0	0	0	0
	0	0	0	0	0	0
	0	0	0	0	0	0
	0	0	0	0	0	0
	0	0	0	0	0	0
	0	0	0	0	0	0
	0	0	0	0	0	0
	0	0	0	0	0	0
	0	0	0	0	0	0
	0	0	0	0	0	0
	0	0	0	0	0	0
	0	0	0	0	0	0

	0	0	0	0	0	0
	0	0	0	0	0	0
	0	0	0	0	0	0
	0	0	0	0	0	0
	0	0	0	0	0	0
	0	0	0	0	0	0
	0	0	0	0	0	0
	0	0	0	0	0	0
	0	0	0	0	0	0
	0	0	0	0	0	0
1	0	0	0	0	1	0
	0	0	0	0	0	0
	0	0	0	0	0	0
	0	0	0	0	0	0
	0	0	0	0	0	0
	0	0	0	0	0	0
	0	0	0	0	0	0
	0	0	0	0	0	0
	0	0	0	0	0	0
	0	0	0	0	0	0
	0	0	0	0	0	0

Przyczyna zgonu 1 udar krwotoczny 2 nagły zgon sercowy	Czas zgonu od zabiegu(dni)	Rehospitalizac ja tak=1 nie=0	Rehospitalizac ja ile dni od zabiegu	Rehospitalizac ja przyczyna 1 udar krwotoczny 2 NZK 3krwiak w miejscu dostepu 4-zawroty glowy, zaburzenia rownowagi	Powikłania odległe tak- 1 nie-0	Skala NIHHS przy ostatniej wizycie w pordanii
		0			0	0
		0			0	0
		0			0	0
		0			0	0
		0			0	1
		0			0	0
		0			0	0
		0			0	0
		0			0	0
		0			0	0
		0			0	3
		0			0	0
		0			0	0
		0			0	0
		0			0	0
		0			0	0
		0			0	0
		0			0	0
		0			0	0
		0			0	0
		0			0	0
		0			0	3
		0			0	2
		0			0	0
		0			0	9
		0			0	0
		0			0	0
		0			0	0
		0			0	6
		0			0	0
		0			0	1
		0			0	0
		0			0	0
		0			0	0

0	0	
		0
0	0	0
0	0	
		0
0	0	
		0
0	0	0
0	0	0
0	0	0
0	0	0
0	0	0
0	0	0
0	0	
		0
0	0	0
0	0	0
0	0	0
0	0	0
0	0	0
0	0	0
0	0	1
0	0	0
0	0	0
0	0	0

Restenoza ICA - poradnia 1 = tak 0 = nie	Przemieszczenie stentu 1 = tak 0=nie poradnia	udar 1 = tak 0=nie	Tia 1= tak 0 = nie	zawał mięśnia sercowego 1 = tak 0=nie	inne powikłanmia 1- jatrogenne uszkodzenie tętnicy żołądkowej lewej i wstrząs krowotoczny 2- krwiak zaotrzewnowy 3 -Zaostrzenie POCHP 4-	Zgon 1 = tak 0=nie
0	0	0	0	0	0	0
0	0	0	0	0	0	0
0	0	0	0	0	0	0
0	0	0	0	0	0	0
0	0	0	0	0	0	0
0	0	0	0	0	0	0
0	0	0	0	0	0	0
0	0	0	0	0	0	0
0	0	0	0	0	0	0
0	0	0	0	0	0	0
0	0	0	0	0	0	0
0	0	0	0	0	0	0
0	0	0	0	0	0	0
0	0	0	0	0	0	0
0	0	0	0	0	0	0
0	0	0	0	0	0	0
0	0	0	0	0	0	0
0	0	0	0	0	0	0
0	0	0	0	0	0	0
0	0	0	0	0	0	0
0	0	0	0	0	0	0
0	0	0	0	0	0	0
0	0	0	0	0	0	0
0	0	0	0	0	0	0
1	0	0	0	0	0	0
0	0	0	0	0	0	0
0	0	0	0	0	0	0
0	0	0	0	0	0	0
0	0	0	0	0	0	0
0	0	0	0	0	0	0
0	0	0	0	0	0	0
0	0	0	0	0	0	0
0	0	0	0	0	0	0
0	0	0	0	0	0	0

0	0	0	0	0	0	0
0	0	0	0	0	0	0
0	0	0	0	0	0	0
0	0	0	0	0	0	0
0	0	0	0	0	0	0
0	0	0	0	0	0	0
0	0	0	0	0	0	0
0	0	0	0	0	0	0
0	0	0	0	0	0	0
0	0	0	0	0	0	0
0	0	0	0	0	1	0
0	0	0	0	0	0	0
0	0	0	0	0	0	0
0	0	0	0	0	0	0
0	0	0	0	0	0	0
0	0	0	0	0	0	0
0	0	0	0	0	0	0
0	0	0	0	0	0	0
0	0	0	0	0	0	0
0	0	0	0	0	0	0
0	0	0	0	0	0	0

Przyczyna zgonu	Czas zgonu od zabiegu(dni)
0	
0	
0	
0	
0	
0	
0	
0	
0	
0	
0	
0	
0	
0	
0	
0	
0	
0	
0	
0	
0	
0	
0	
0	
0	
0	
0	
0	
0	
0	
0	
0	
0	
0	

0

0

0

0

0

0

0

0

0

0

0

0

0

0

0

0

0

0

0

0

0

[redacted]

[redacted]

[redacted]

[redacted]

[redacted]

[redacted]

[redacted]

[redacted]

[redacted]

[redacted]

[redacted]

[redacted]

[redacted]

[redacted]

[redacted]

[redacted]

[redacted]

[redacted]

[redacted]

[redacted]

[redacted]

[redacted]

[redacted]

[redacted]

[redacted]

[redacted]

[redacted]

[redacted]

[redacted]

[redacted]

[redacted]
